# CRISPR/Cas9-Mediated Knock-Out of the *MtCLE35* Gene Highlights Its Key Role in the Control of Symbiotic Nodule Numbers under High-Nitrate Conditions

**DOI:** 10.3390/ijms242316816

**Published:** 2023-11-27

**Authors:** Maria A. Lebedeva, Daria A. Dobychkina, Lyudmila A. Lutova

**Affiliations:** Department of Genetics and Biotechnology, Saint Petersburg State University, Universitetskaya Emb. 7/9, 199034 Saint Petersburg, Russia; dobichkinadaria@gmail.com (D.A.D.); l.lutova@spbu.ru (L.A.L.)

**Keywords:** autoregulation of nodulation (AON), CLE peptides, rhizobia, nodulation, nitrate

## Abstract

Legume plants have the ability to establish a symbiotic relationship with soil bacteria known as rhizobia. The legume–rhizobium symbiosis results in the formation of symbiotic root nodules, where rhizobia fix atmospheric nitrogen. A host plant controls the number of symbiotic nodules to meet its nitrogen demands. CLE (CLAVATA3/EMBRYO SURROUNDING REGION) peptides produced in the root in response to rhizobial inoculation and/or nitrate have been shown to control the number of symbiotic nodules. Previously, the *MtCLE35* gene was found to be upregulated by rhizobia and nitrate treatment in *Medicago truncatula*, which systemically inhibited nodulation when overexpressed. In this study, we obtained several knock-out lines in which the *MtCLE35* gene was mutated using the CRISPR/Cas9-mediated system. *M. truncatula* lines with the *MtCLE35* gene knocked out produced increased numbers of nodules in the presence of nitrate in comparison to wild-type plants. Moreover, in the presence of nitrate, the expression levels of two other nodulation-related *MtCLE* genes, *MtCLE12* and *MtCLE13*, were reduced in rhizobia-inoculated roots, whereas no significant difference in *MtCLE35* gene expression was observed between nitrate-treated and rhizobia-inoculated control roots. Together, these findings suggest the key role of *MtCLE35* in the number of nodule numbers under high-nitrate conditions, under which the expression levels of other nodulation-related *MtCLE* genes are reduced.

## 1. Introduction

Legume plants establish a symbiotic relationship with gram-negative soil bacteria, collectively called rhizobia, which results in the development of nitrogen-fixing nodules on the host plant roots. A host plant controls the number of symbiotic nodules to meet its nitrogen demands through a system known as AON (autoregulation of nodulation, reviewed in [1]). Key players of AON are CLE (CLAVATA3/EMBRYO SURROUNDING REGION) peptides produced in the root in response to rhizobial inoculation and/or nitrate to control the number of symbiotic nodules. The CLE peptides produced in the root are transported through the xylem to the shoot, where they are recognized by their receptors, LRR family receptor kinases [2,3,4]. As a result, a shoot-derived signaling pathway is induced to inhibit subsequent nodulation on the roots [5].

In *Medicago truncatula*, three *MtCLE* genes have been reported to act as negative regulators of symbiotic nodule development [6,7,8,9]. Among them, *MtCLE12* and *MtCLE13* genes are upregulated in response to rhizobia inoculation, and overexpression of these genes inhibits nodulation in a systemic manner [6]. The symbiosis-specific transcription factor, NIN (NODULE INCEPTION), is suggested to be responsible for the activation of *CLE* genes in response to nodulation [10,11]. It has been shown that NIN directly binds to the promoter of *MtCLE13* and activates its expression, thereby triggering a feedback regulatory mechanism to limit the number of symbiotic nodules [11]. In addition to the *MtCLE12* and *MtCLE13* genes, the *MtCLE35* gene has been identified as an inhibitor of nodulation, which is activated by both rhizobia and nitrate treatment [7,8,9]. The expression levels of *MtCLE35* are upregulated in the roots in response to nitrate addition, whereas *MtCLE12* and *MtCLE13* genes are not activated by nitrate [7]. Nitrate-induced activation of *MtCLE35* expression is mediated by the NLP1 (NIN-LIKE PROTEIN 1) transcription factor, which acts as a regulator of the nitrate response in *M. truncatula*. Additionally, a direct binding of NLP1 to the *MtCLE35* promoter has been reported [12]. Rhizobia-induced *MtCLE35* expression has been shown to be dependent on the NIN transcription factor [12]. Therefore, the expression of the *MtCLE35* gene is regulated by two transcription factors, NIN and NLP1, in response to rhizobia and nitrate treatment, respectively, and this gene is suggested to mediate the inhibition of nodulation through AON in response to nitrate treatment.

In this study, to further elucidate the role of the *MtCLE35* gene in nodulation, we used CRISPR/Cas9-mediated gene editing to obtain *MtCLE35* knock-out lines. Lines with the *MtCLE35* gene knocked out produced increased numbers of nodules in the presence of nitrate in comparison to the parental wild-type line, R108. Moreover, we found that in the presence of 10 mM KNO_3_, the expression levels of two other nodulation-related *MtCLE* genes, *MtCLE12* and *MtCLE13*, were reduced in rhizobia-inoculated roots; however, no significant changes in *MtCLE35* gene expression were observed. These findings highlight the key role of *MtCLE35* in the inhibition of nodulation under high-nitrate conditions when two other nodulation-related *MtCLEs*, *MtCLE12* and *MtCLE13*, are downregulated.

## 2. Results

### 2.1. Generation of Medicago Truncatula Lines through the CRISPR/Cas9-Mediated Knock-Out of the MtCLE35 Gene

#### 2.1.1. Construction of the Vector for the CRISPR/Cas9-Mediated Editing of the *MtCLE35* Gene and Stable Transformation of *Medicago truncatula* Plants

The target sequence for the CRISPR/Cas9-mediated editing of the *MtCLE35* gene (19 bp sequence, 187–205 bp within the CDS of the *MtCLE35* gene, Supplementary Figure S1A) was ligated into the pHSE401 vector. The resulting construct was checked via sequencing and then introduced into *Agrobaterium tumefaciens* strain AGL1 for the subsequent stable transformation of the *M. truncatula* leaf explants, according to Cosson et al., 2006 [13]. As a result, 20 T0 plants regenerated from the transgenic callus were selected and subsequently checked for mutations in the *MtCLE35* gene.

#### 2.1.2. Genotyping of T0–T1 Plants

**Figure 1:**
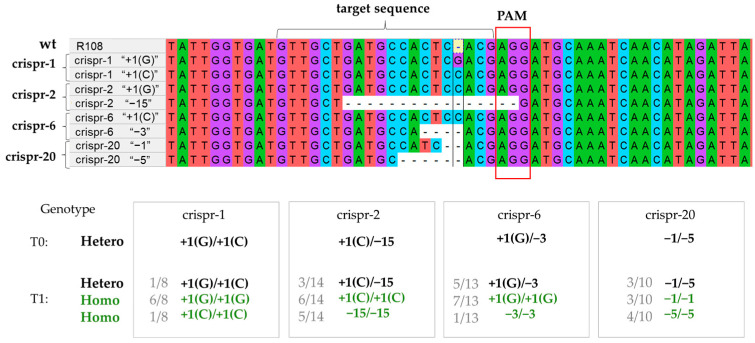
Alleles of the *MtCLE35* gene identified in crispr-1, crispr-2, crispr-6 and cripr-20 plants due to the CRISPR/Cas9-mediated editing of the *MtCLE35* gene.. In nucleotides sequences, each color represents a different nucleotide (A, T, C or G). PAM (protospacer adjacent motif) is shown with a red box. The target sequence is shown with a bracket. Low panel, genotypes of T0 and T1 plants: “+1” indicates 1-bp insertion within the *MtCLE35* CDS, whereas −1, −5 and −15 correspond to deletions of 1, 5 and 15 nucleotides, respectively. For T1 plants, fractional numbers indicate the fraction of each genotype observed among the total number of progenies derived from the same T0 plant. “Hetero” and “homo” correspond to heterozygotes and homozygotes, respectively.

We analyzed the nucleotide sequences of the *MtCLE35* gene in the T0 plants via sequencing. Analysis of the sequencing chromatogram using the Synthego ICE program (https://ice.synthego.com/#/, accessed on 11 July 2023) revealed deletions of varying lengths within the CDS of the *MtCLE35* gene, as well as the insertion of a single nucleotide in several plants (Supplementary Table S1). For further studies, the progeny of four plants was obtained (crispr-1, crispr-2, crispr-6 and cripr-20), and both homozygous and heterozygous plants were identified among the progeny of these plants (Figure 1). Homozygous plants with loss-of-function mutations in the *MtCLE35* gene were selected for further analysis (T1 progeny derived from crispr-1 and crispr-6 plants), and T3 progeny of these plants were used to estimate the effect of *MtCLE35* knock-out on nodulation. These lines were designated as “crispr-1” and “crispr-6”. Both these lines possess 1-bp insertion (“C” in the case of “crispr-1” and “G” in the case of “crispr-6”), which occurred three bp upstream of the PAM sequence (after 202 bp in the *MtCLE35* CDS); this was confirmed through sequencing the *MtCLE35* gene in the T1 and T3 plants. Such 1-bp insertion should result in a frameshift, leading to the occurrence of a premature stop codon and the translation of truncated protein lacking the CLE domain sequence (Supplementary Figure S1B).

### 2.2. Effect of MtCLE35 Knock-Out on Nodule Number under Nitrogen-Free and Nitrate Treatment Conditions

**Figure 2:**
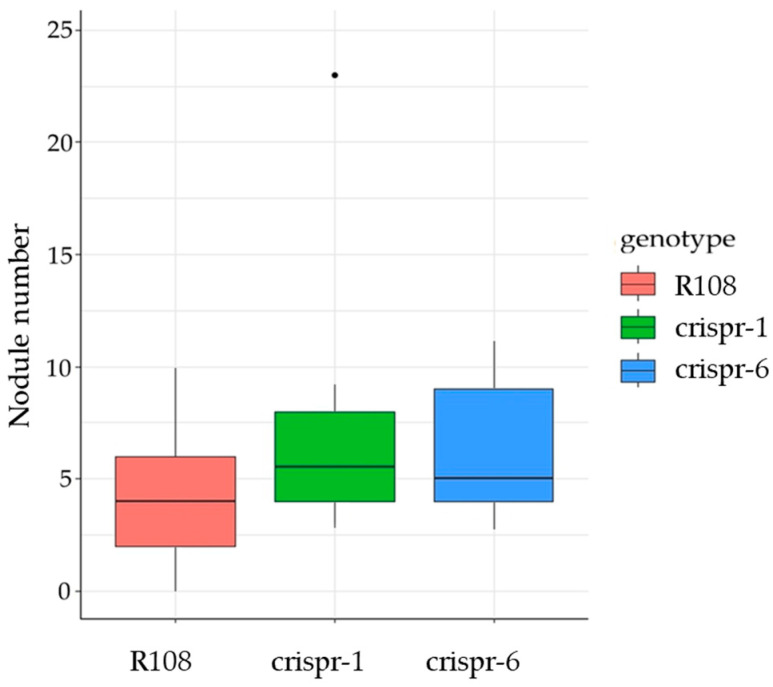
Box plots showing the number of nodules at 28 dpi (days post inoculation) in wild-type (R108) and *MtCLE35* knock-out (crispr-1 and crispr-6) plants grown without nitrate addition. No statistical difference was found in the nodule numbers of these three genotypes (Wilcoxon test, n = 10–16).

To evaluate the effect of loss-of-function mutations in the *MtCLE35* gene on nodulation, T3 homozygous plants (“crispr-1” and “crispr-6” lines) were used. The number of nodules was estimated four weeks after inoculation with rhizobia in “crispr-1” and “crispr-6” lines and compared to the R108 parental line under nitrogen-free conditions. We expected that knock-out of the *MtCLE35* gene, which is known as a negative regulator of symbiosis, could result in increased nodule numbers. However, we found no statistically significant differences between the number of nodules formed in the *MtCLE35* knock-out plants (“crispr-1” and “crispr-6” lines) and the R108 control plants (Figure 2). This result was reproduced in three independent experiments.

**Figure 3:**
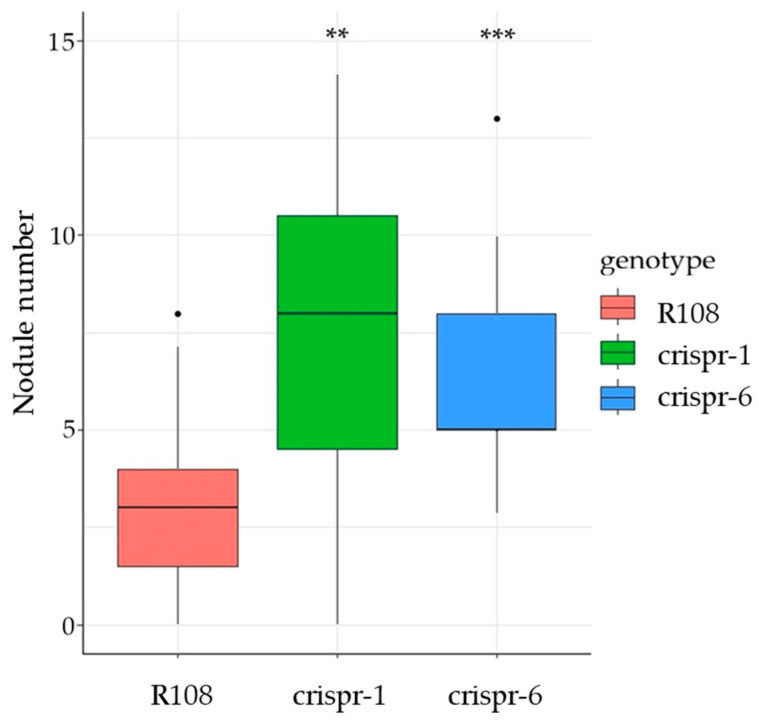
Box plots showing the number of nodules at 28 dpi in wild-type (R108) and *MtCLE35* knock-out (crispr-1 and crispr-6) plants grown in the presence of 10 mM KNO_3_. ** *p* < 0.01, *** *p* < 0.001 (Wilcoxon test, n = 15–19).

Next, to study whether the loss of the *MtCLE35* gene function affects the ability of plants to suppress the development of symbiotic nodules in the presence of nitrate, we estimated the number of nodules of “crispr-1”, “crispr-6” and R108 plants grown in the presence of 10 mM KNO_3_. As we reported previously, treatment of *M. truncatula* plants with 10 mM KNO_3_ induced *MtCLE35* expression in the roots [7]. Moreover, treatment with this concentration of KNO_3_ suppressed nodulation in R108 plants grown in aeroponic systems (Supplementary Figure S2). Under nitrate treatment, the number of nodules was significantly increased in the *MtCLE35* knock-out, “crispr-1” and “crispr-6” plants, in comparison to the R108 control plants (Figure 3). Thus, the loss of the *MtCLE35* function resulted in an increased number of symbiotic nodules in the presence of 10 mM KNO_3_.

### 2.3. Effect of Nitrate on the Expression Levels of MtCLE Genes in Rhizobia-Inoculated Roots

Among nodulation-related *MtCLE* genes, only the *MtCLE35* gene is activated by nitrate, whereas the expression levels of two other *MtCLE*s, *MtCLE12* and *MtCLE13*, do not increase in response to nitrate treatment [7]. Here, we found statistically significant differences in nodule numbers between the R108 and MtCLE35-crispr plants under high nitrate, indicating that MtCLE35 plays a key role in the control of nodule numbers in the presence of nitrate. We suggested that that the expression levels of two other *MtCLE* genes, *MtCLE12* and *MtCLE13*, could be downregulated in nodulating roots under nitrate addition so that their expression levels are not high enough to activate AON in the presence of nitrate.

**Figure 4:**
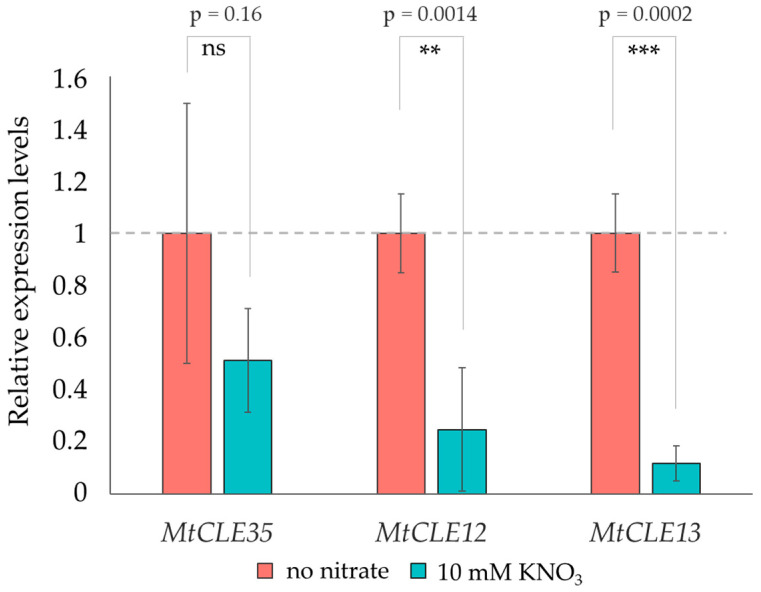
Expression levels of *MtCLEs* in the roots after inoculation with rhizobia at 7 dpi; plants were grown in nitrogen-free (no nitrate) media or in the presence of 10 mM KNO_3_. Relative expression levels were normalized to 1, relative to the control plants grown without N (no nitrate), as indicated by the dotted lines. Results are mean ± SD of 5 biological repeats. Asterisks mark statistically significant differences (ns—not significant, ** *p* < 0.01; *** *p* < 0.001, Student *t*-test, n = 5). *p*-values are indicated above the respective comparison.

To check this, we analyzed the expression levels of the *MtCLE12* and *MtCLE13* genes at 7 dpi (days post inoculation) in the roots of rhizobia-inoculated R108 plants grown in the presence of 10 mM KNO_3_ and R108 plants grown without nitrate addition. We found no statistical differences in *MtCLE35* expression levels between the control and nitrate-treated plants; however, the expression levels of *MtCLE12* and *MtCLE13* were significantly reduced in the inoculated roots of nitrate-treated plants in comparison with control plants grown without nitrate (Figure 4).

## 3. Discussion

In this study, we obtained several lines with CRISPR/Cas9-mediated knock-out of the *MtCLE35* gene. T0 plants, obtained as a result of *A. tumefaciens*-mediated transformation, from which these lines were derived, appeared to be heterozygotes with both alleles of the *MtCLE35* gene mutated due to gene editing. Among the T1 progeny, homozygous plants carrying loss-of-function mutations in the *MtCLE35* gene were selected.

We estimated the number of symbiotic nodules in T3 plants with *MtCLE35* knocked out and found statistically significant differences in the nodule numbers of R108 and MtCLE35-crispr plants under high nitrate, whereas without nitrate the number of symbiotic nodules in *MtCLE35* knock-out plants did not differ from that in R108 plants. This result is consistent with the data obtained by Moreau et al. [9], who showed that MtCLE35 RNAi in transgenic roots resulted in increased nodule numbers in the presence of high-nitrate conditions (10 mM NH_4_NO_3_).

**Figure 5:**
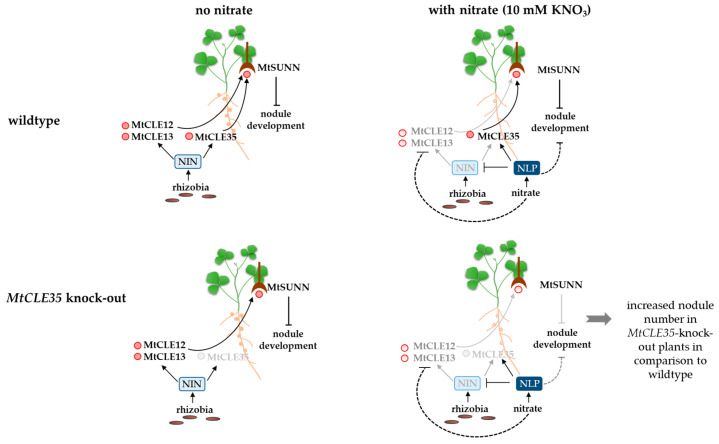
A model explaining the increased number of nodules found in *MtCLE35* knock-out plants in comparison to wild-type plants under high-nitrate conditions. The dotted line indicates an indirect effect. In the absence of nitrate (left panels), legume–rhizobia interaction results in the formation of symbiotic nodules. Rhizobia induce a signaling cascade leading to the activation of key regulators of symbiosis. Among them, the expression of the *NIN* gene is induced, which encodes a key transcription factor regulating both rhizobia infection and nodule primordium development. The NIN transcription factor activates the expression of the *CLE* genes in response to rhizobia inoculation, including *MtCLE12, MtCLE13* and *MtCLE35*. As a result, the AON system is activated by root-to-shoot transported MtCLE peptides, activating the MtSUNN receptor kinase, which operates in the phloem cells of leaves. In turn, a shoot-derived signaling pathway is induced to inhibit nodulation via a negative feedback mechanism. Knock-out of the *MtCLE35* gene does not significantly reduce the induction of AON since MtCLE35 acts redundantly with MtCLE12 and MtCLE13 to inhibit nodulation, and, therefore, the nodule number is not significantly increased in *MtCLE35* knock-out plants. The presence of nitrate (left panels) downregulates symbiotic nodulation. One of the known mechanisms of such inhibition is mediated by the nitrate-activated NLP1 transcription factor, which has been suggested to inhibit nodulation by interfering with the NIN action. As a result, the expression levels of NIN-target genes, including *MtCLE12* and *MtCLE13*, are decreased. However, the expression of the *MtCLE35* gene is not significantly reduced in developing nodules under high-nitrate conditions since the nitrate-activated NLP1 transcription factor is able to induce *MtCLE35* expression. Therefore, in the presence of nitrate, MtCLE35 acts as the key inductor of AON since the expression levels of two other *MtCLE* genes, *MtCLE12* and *MtCLE13*, are significantly decreased. Thereby, knock-out of the *MtCLE35* gene results in a significantly increased nodule number in the presence of nitrate.

Therefore, knock-out of the *MtCLE35* gene resulted in increased numbers of nodules only under high-nitrate conditions, whereas in the absence of nitrate, the loss of *MtCLE35* function did not lead to increased nodule numbers. The possible explanation for this could be that in the absence of nitrate, two other MtCLEs, MtCLE12 and MtCLE13, act redundantly with MtCLE35 to induce a feedback inhibitory effect on nodule number. However, in the presence of high amounts of nitrate, the *MtCLE12* and *MtCLE13* genes are downregulated, and, therefore, the MtCLE35 peptide becomes the key regulator of the nodule number (Figure 5).

Indeed, according to expression analysis, the *MtCLE12* and *MtCLE13* genes are significantly downregulated in rhizobia-inoculated roots in the presence of nitrate, whereas the expression levels of the *MtCLE35* gene did not change significantly. We can speculate that the downregulation of *MtCLE12* and *MtCLE13* could be mediated by the NLP1 transcription factor, which is known to be activated by nitrate and is suggested to form heterodimers with NIN transcription factor to interfere with its action, and thereby inhibit the expression of NIN target genes [14]. The activation of *MtCLE12* and *MtCLE13* expression in nodulating roots is NIN-dependent [6]; moreover, the direct binding of NIN to the *MtCLE13* promoter has been shown [11]. In contrast to *MtCLE12* and *MtCLE13*, the promoter of the *MtCLE35* gene contains both NIN and NLP1 binding sites [9,12], and therefore, its expression could be activated by both NIN TF in response to rhizobia and by NLP1 TF in response to nitrate. Nitrate induces the activation of the NLP1 transcription factor, which mediates the inhibition of NIN-regulated genes, thereby suppressing nodulation. In consistence with this, the NIN target genes, *MtCLE13* and *MtCLE12*, are downregulated in nodulating roots in response to nitrate treatment (see Figure 5). Previously, it was shown that the *MtCLE35* gene is activated in the root by nitrate addition under non-symbiotic conditions [7,8,9], and the NLP1 transcription factor was shown to activate *MtCLE35* in response to nitrate [9,12]. Under symbiotic conditions, NIN activates the expression of the *MtCLE35* gene, and we can speculate that NIN-dependent activation of the *MtCLE35* is diminished in response to nitrate in nodulating roots, as observed for *MtCLE12* and *MtCLE13*. However, since the *MtCLE35* gene is positively regulated, not only by NIN but also by NLP1, which is activated under the presence of nitrate, the overall expression level of *MtCLE35* is not decreased significantly in nitrate-treated developing nodules, notwithstanding the reduction of NIN-dependent transcriptional activation. Therefore, MtCLE35 becomes the key regulator of the nodule number under high-nitrate conditions, while *MtCLE12* and *MtCLE13* expression levels are downregulated (see Figure 5).

Therefore, our data highlight the key role of MtCLE35 in the control of nodule numbers under high-nitrate conditions and elucidate the complex regulation of *MtCLE* genes via nitrate, which had evolved to control the number of symbiotic nodules. The *M. truncatula* lines with CRISPR/Cas9-mediated knock-out of the *MtCLE35* gene have an increased number of nodules in the presence of nitrate, which should result in the increase of their overall symbiotic efficiency in comparison to the wild-type plants. This strategy could be tested in other legumes in order to produce legume crops with increased nodulation ability in nitrate-polluted soils.

## 4. Materials and Methods

### 4.1. Plant Material and Growth Conditions

*M. truncatula* seeds (R108, “crispr-1” and “crispr-6” *MtCLE35* knock-out lines) were sterilized with sulfuric acid for 8 min, washed 10 times with sterile distilled water, and then were germinated on plates with 1% agar. The plates were kept at +4 °C for 7–10 days and then were kept for 48 h at room temperature in a dark place for germination. For the nodulation experiments, plants were grown on Petri dishes using Fahraeus medium [15] either without nitrate or with the addition of 10 mM KNO_3_ (for nitrate treatment) for 7 days after germination and then were transferred into an aeroponic system containing either nitrogen-free aeroponic media [16] or aeroponic media supplied with 10 mM KNO_3_. The plants were grown under a 16-h photoperiod at 21 °C. In 3–4 days, the plants were inoculated with *Sinorhizobium meliloti* strain 2011 through the addition of a fresh bacterial culture to the aeroponic media (with a final OD_600_ concentration of approx. 0.005). The number of nodules on the roots was calculated at 28 days after rhizobia inoculation. For the gene expression analysis, the roots of the R108 plants, grown as described above, with either no nitrate or in the presence of 10 mM KNO_3,_ were harvested at 7 dpi.

### 4.2. Construction of the Vector for the CRISPR/Cas9-Mediated Knock-out of the MtCLE35 Gene

The target sequence of the *MtCLE35* gene was selected using CRISPOR software (http://crispor.tefor.net/, accessed on 11 September 2021) [17] and checked for possible off-targets using the Cas-OFFinder algorithm (http://www.rgenome.net/cas-offinder/, accessed on 11 September 2021) [18]. Oligonucleotides (CLE35_target_F: ATTGGTTGCTGATGCCACTCACG and CLE35_target_R: AAACCGTGAGTGGCATCAGCAAC, 5′-overhangs are underlined) were synthesized by Evrogen Inc. (Russia, Moscow), and cloned into BsaI-digested pHSE401 vectors [19] according to the protocol described by Xing et al. [19]. The insertion of the target sequence was checked via sequencing. The obtained vector was introduced into the *Agrobacterium tumefaciens* strain AGL1.

### 4.3. Agrobacterium Tumefaciens-Mediated Transformation 

*Agrobacterium tumefaciens*-mediated transformation of leaf explants of the R108 line was performed as described by Cosson et al. and Tvorogova et al. [13,20]. T0 plants, regenerated via transgenic callus, were first transferred to the plates with Fahraeus media [15], then after the development of several leaves and roots, to pots filled with soil.

### 4.4. Genotyping of T0–T1 Plants

Nucleotide sequences of the *MtCLE35* gene in *Medicago truncatula* were analyzed via the sequencing of PCR products amplified using primers specific for *MtCLE35* CDS (MtCLE35-FOR: ATGGCAAACACACAAATAACTATATTT; MtCLE35-REV: CTACTTGTTTTGTGGACCTGCA). Genomic DNA was extracted from the plant leaves using the Edwards buffer [21]. Alternatively, the NucleoType Plant PCR kit (Macherey-Nagel, Düren, Germany) was used for the rapid genotyping of T0 and T1 plants, according to the manufacturer’s protocol. The obtained chromatograms corresponding to the *MtCLE35* gene sequence in T0 and T1 plants were analyzed using the Synthego ICE program (https://ice.synthego.com/#/, accessed on 11 July 2023) to identify possible deletions or insertions of nucleotides.

### 4.5. RNA Extraction and Quantitative Reverse Transcription PCR (qRT-PCR) Analysis

The Total RNA was extracted from the plant roots using TRIZol reagent according to the manufacturer’s instructions (Thermo Scientific, Waltham, MA, USA). A Rapid Out DNA Removal Kit (Thermo Fisher Scientific, Waltham, MA, USA) was used to remove DNA from the RNA samples. A NanoDrop 2000c UV-Vis Spectrophotometer (Thermo Scientific, Waltham, MA, USA) was used to measure the RNA concentration and quality.

500 ng of extracted RNA was used for cDNA synthesis in each sample. The cDNA was synthesized using the Revert Aid Reverse Transcriptase kit (Thermo Fisher Scientific, Waltham, MA, USA). Also, qRT–PCR experiments were conducted with a CFX-96 real-time PCR detection system with a C1000 thermal cycler (Bio-Rad Laboratories, Hercules, CA, USA) using Eva Green intercalating dyes (Synthol, Moscow, Russia). The data were analyzed by the CFX Manager software 2.1 version 2.1.1022.0523 (Bio-Rad Laboratories, Alfred Nobel Drive, Hercules, CA, USA) with the 2^−ΔΔCt^ method [22]. Actin (Medtr7g026230) and ubiquitin (Medtr4g091580) genes were used as reference genes, and qRT–PCR reactions were run in three replicates. The specificity of the PCR amplification was confirmed based on the dissociation curve (55–95 °C). The primers were synthesized by Evrogen (Evrogen, Moscow, Russia). The primers used for qRT–PCR are listed in Supplementary Table S2. The primers for the reference genes and the *MtCLE35* gene were taken from Lebedeva et al. [7], and primers specific to the *MtCLE12* and *MtCLE13* genes were taken from Mortier et al. [6].

### 4.6. Statistical Methods

In the gene expression assay, five biological repeats per variant were used in each experiment. A Student t-test was used to compare gene expression levels. The box plots illustrating nodule numbers were drawn in RStudio (https://rstudio.com/, accessed on 11 September 2023, R version 4.2.3), and a Wilcoxon test was used to compare the number of symbiotic nodules. Nodulation assays were performed during three independent experiments, 10–19 plants in each experimental group (see details in Figure 2, Figure 3 and Figure 4 captures).

## Data Availability

All the data obtained are contained within the article and Supplementary Materials.

## Supplementary Materials

The following supporting information can be downloaded at: https://www.mdpi.com/article/10.3390/ijms242316816/s1.

## References

[1] Li Y., Pei Y., Shen Y., Zhang R., Kang M., Ma Y., Li D., Chen Y. (2022). Progress in the Self-Regulation System in Legume Nodule Development-AON (Autoregulation of Nodulation). Int. J. Mol. Sci..

[2] Searle I.R., Men A.E., Laniya T.S., Buzas D.M., Iturbe-Ormaetxe I., Carroll B.J., Gresshoff P.M. (2003). Long-Distance Signaling in Nodulation Directed by a CLAVATA1-like Receptor Kinase. Science.

[3] Nishimura R., Hayashi M., Wu G.-J., Kouchi H., Imaizumi-Anraku H., Murakami Y., Kawasaki S., Akao S., Ohmori M., Nagasawa M. (2002). HAR1 Mediates Systemic Regulation of Symbiotic Organ Development. Nature.

[4] Okamoto S., Ohnishi E., Sato S., Takahashi H., Nakazono M., Tabata S., Kawaguchi M. (2009). Nod Factor/Nitrate-Induced CLE Genes that Drive HAR1-Mediated Systemic Regulation of Nodulation. Plant Cell Physiol..

[5] Reid D.E., Ferguson B.J., Hayashi S., Lin Y.-H., Gresshoff P.M. (2011). Molecular Mechanisms Controlling Legume Autoregulation of Nodulation. Ann. Bot..

[6] Mortier V., Den Herder G., Whitford R., Van de Velde W., Rombauts S., D’haeseleer K., Holsters M., Goormachtig S. (2010). CLE Peptides Control Medicago Truncatula Nodulation Locally and Systemically. Plant Physiol..

[7] Lebedeva M., Azarakhsh M., Yashenkova Y., Lutova L. (2020). Nitrate-Induced CLE Peptide Systemically Inhibits Nodulation in Medicago Truncatula. Plants.

[8] Mens C., Hastwell A.H., Su H., Gresshoff P.M., Mathesius U., Ferguson B.J. (2021). Characterisation of Medicago Truncatula CLE34 and CLE35 in Nitrate and Rhizobia Regulation of Nodulation. New Phytol..

[9] Moreau C., Gautrat P., Frugier F. (2021). Nitrate-Induced CLE35 Signaling Peptides Inhibit Nodulation through the SUNN Receptor and miR2111 Repression. Plant Physiol..

[10] Soyano T., Hirakawa H., Sato S., Hayashi M., Kawaguchi M. (2014). NODULE INCEPTION Creates a Long-Distance Negative Feedback Loop Involved in Homeostatic Regulation of Nodule Organ Production. Proc. Natl. Acad. Sci. USA.

[11] Laffont C., Ivanovici A., Gautrat P., Brault M., Djordjevic M.A., Frugier F. (2020). The NIN Transcription Factor Coordinates CEP and CLE Signaling Peptides That Regulate Nodulation Antagonistically. Nat. Commun..

[12] Luo Z., Lin J., Zhu Y., Fu M., Li X., Xie F. (2021). NLP1 Reciprocally Regulates Nitrate Inhibition of Nodulation through SUNN-CRA2 Signaling in Medicago Truncatula. Plant Commun..

[13] Cosson V., Durand P., d’Erfurth I., Kondorosi A., Ratet P., Wang K. (2006). Medicago Truncatula Transformation Using Leaf Explants. Agrobacterium Protocols.

[14] Lin J., Li X., Luo Z., Mysore K.S., Wen J., Xie F. (2018). NIN Interacts with NLPs to Mediate Nitrate Inhibition of Nodulation in Medicago Truncatula. Nat. Plants.

[15] Fahraeus G. (1957). The Infection of Clover Root Hairs by Nodule Bacteria Studied by a Simple Glass Slide Technique. J. Gen. Microbiol..

[16] Lullien V., Barker D.G., de Lajudie P., Huguet T. (1987). Plant Gene Expression in Effective and Ineffective Root Nodules of Alfalfa (*Medicago Sativa*). Plant Mol. Biol..

[17] Concordet J.-P., Haeussler M. (2018). CRISPOR: Intuitive Guide Selection for CRISPR/Cas9 Genome Editing Experiments and Screens. Nucleic Acids Res..

[18] Cas-OFFinder: A Fast and Versatile Algorithm that Searches for Potential Off-Target Sites of Cas9 RNA-Guided Endonucleases|Bioinformatics|Oxford Academic. https://academic.oup.com/bioinformatics/article/30/10/1473/267560.

[19] Xing H.-L., Dong L., Wang Z.-P., Zhang H.-Y., Han C.-Y., Liu B., Wang X.-C., Chen Q.-J. (2014). A CRISPR/Cas9 Toolkit for Multiplex Genome Editing in Plants. BMC Plant Biol..

[20] Tvorogova V.E., Fedorova Y.A., Potsenkovskaya E.A., Kudriashov A.A., Efremova E.P., Kvitkovskaya V.A., Wolabu T.W., Zhang F., Tadege M., Lutova L.A. (2019). The WUSCHEL-Related Homeobox Transcription Factor MtWOX9-1 Stimulates Somatic Embryogenesis in Medicago Truncatula. Plant Cell Tissue Organ. Cult..

[21] Edwards A., Civitello A., Hammond H.A., Caskey C.T. (1991). DNA Typing and Genetic Mapping with Trimeric and Tetrameric Tandem Repeats. Am. J. Hum. Genet..

[22] Livak K.J., Schmittgen T.D. (2001). Analysis of relative gene expression data using real-time quantitative PCR and the 2(-Delta Delta C(T)) method. Methods.

