# Supplementary material for: CRISPR/Cas9-Mediated Knock-Out of the MtCLE35 Gene Highlights Its Key Role in the Control of Symbiotic Nodule Numbers under High-Nitrate Conditions
Source: Int J Mol Sci. 2023 Nov 27;24(23):16816. doi: 10.3390/ijms242316816 (PMC10706395; doi:10.3390/ijms242316816)
Supplement: Supplementary file 1 [file ijms-24-16816-s001.zip › ijms-2697537-supplementary.pdf]

International Journal of *Molecular Sciences*

MDPI

A

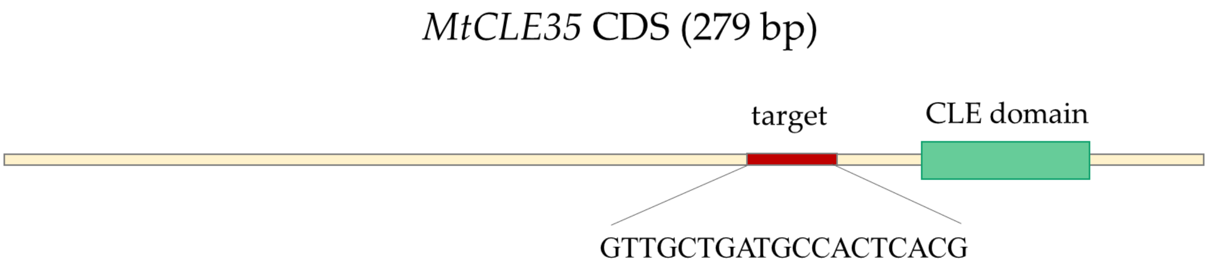

B

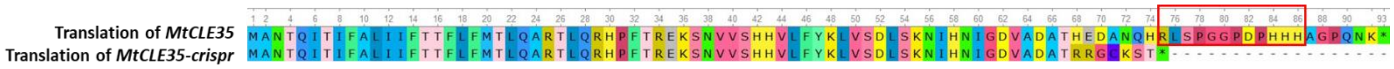

Supplementary Figure S1. Target sequence selected for CRISPR/Cas9-mediated editing of the MtCLE35 gene (A); and the comparison of amino acid sequences of the wild-type MtCLE35 protein and the MtCLE35-crispr protein resulted from the translation of the MtCLE35 allele with 1-bp insertion found in "crispr-1" and "crispr-6" lines (B). CLE domain sequence is marked with a red box.

**T0 plant ID (№/sample ID)**		**In/del**
1	#1	+1(G)/ +1(C)
2	#2	-15/+1
3	#4	deletions of various length
4	#6	+1/-3
5	#8	deletions of various length
6	#9	-4/-3
7	#10	-15
8	#11	-20/-1
9	#12	-4/-5
10	#13	+1/-2
11	#14	deletions of various length
12	#15	-1/-5
13	#16	0 (wild type)
14	#17	+1(G)/ +1(C)
15	#18	-20/-1
16	#19	0 (wild type)
17	#20	-1/-5
18	#21	+1/-2
19	#25	-5/-1
**20**	**#27**	**-20/-1**

Supplementary Table S1. Genotypes of T0 plants obtained after transformation with the construct for CRISPR/Cas9-mediated editing of the *MtCLE35* gene. The data are obtained based on the analysis of sequencing chromatogram for the *MtCLE35* gene using Synthego ICE tool. For two plants, #16 and #19, no editing events were found (wild type genotype). In/del – insertions and deletions within the *MtCLE35* CDS, negative numbers correspond to the length of deletions in bp, +1 corresponds to 1 bp insertion. Three plants (#4, #8, #14) had deletions of various length according to Synthego ICE analysis indicating multiallelic changes in the *MtCLE35* gene in these plants.

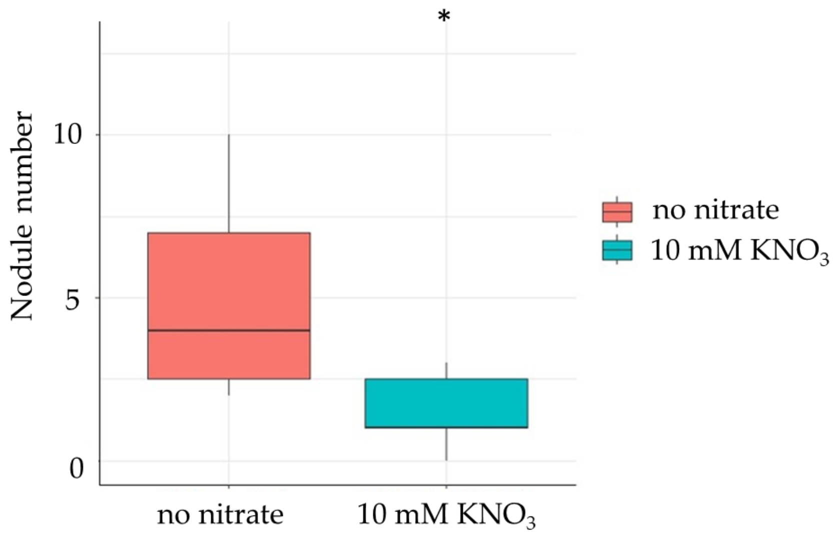

Supplementary Figure S2. Number of nodules at 28 dpi in wild-type plants (R108) grown without nitrate and in presence of 10 mM KNO_3. * $p<0.05$ (wilcoxon test, n=15).
